# The 1,3-Dioctadecyl-1*H*-imidazol-3-ium Based Potentiometric Surfactant Sensor for Detecting Cationic Surfactants in Commercial Products

**DOI:** 10.3390/s22239141

**Published:** 2022-11-25

**Authors:** Nikola Sakač, Dubravka Madunić-Čačić, Dean Marković, Bartolomeo Della Ventura, Raffaele Velotta, Anita Ptiček Siročić, Brunislav Matasović, Nikolina Sermek, Bojan Đurin, Bojan Šarkanj, Marija Jozanović

**Affiliations:** 1 Faculty of Geotechnical Engineering, University of Zagreb, 42000 Varaždin, Croatia; 2 Saponia Chemical, Pharmaceutical and Foodstuff Industry, Inc., 31000 Osijek, Croatia; 3 Department of Biotechnology, University of Rijeka, 51000 Rijeka, Croatia; 4 Department of Physics “E. Pancini”, Università Di Napoli Federico II, 80126 Napoli, Italy; 5 Department of Chemistry, University of Osijek, 31000 Osijek, Croatia; 6 Department of Civil Engineering, University North, 42000 Varaždin, Croatia; 7 Department of Food Technology, University North, 48000 Koprivnica, Croatia

**Keywords:** cationic surfactant, ionophore, disinfectant, antiseptic, potentiometry, sensor

## Abstract

A low-cost and fast potentiometric surfactant sensor for cationic surfactants, based on the new ion-pair 1,3-dioctadecyl-1*H*-imidazol-3-ium-tetraphenylborate (DODI-TPB), is presented. The new cationic surfactant DODI-Br was synthesized and characterized by NMR, LC-MS, and elemental analysis, and was used for synthesis of the DODI-TPB ionophore. The DODI-TPB surfactant sensor was obtained by implementation of the ionophore in PVC. The sensor showed excellent response characteristics with near-Nernstian slopes to the cationic surfactants DMIC, CPC, CTAB, and Hyamine 1622. The highest voltage responses were obtained for DMIC and CPC (58.7 mV/decade of activity). DMIC had the lowest detection limit (0.9 × 10^−6^ M) and the broadest useful linear concentration range (1.8 × 10^−6^ to 1.0 × 10^−4^ M). An interference study showed remarkable stability. Potentiometric titration curves for the titration of cationic surfactants (DMIC, CPC, CTAB, and Hyamine 1622), with DDS and TPB used as titrants, showed sigmoidal curves with well-defined inflexion points and a broad signal change. The standard addition method was successfully applied with recovery rates from 98.9 to 101.2 at two concentrations. The amount of cationic surfactant found in disinfectants and antiseptics was in good agreement with the referent two-phase titration method and the surfactant sensor on the market. This new surfactant sensor represents a low-cost alternative to existing methods for cationic surfactant detection.

## 1. Introduction

During the global COVID-19 pandemic, cationic surfactants have been widely used as antiseptics and disinfectants. Recent studies revealed that the growth in production and usage of surfactants was +196%, for biocides it was +152%, and for cationic quaternary ammonium surfactants (used as surfactants and biocides) it was +331% [1]. Surfactants represent a group of organic molecules that lower the surface tension. They consist of a charged head and a long alkyl chain (“fat hydrophobic chain”). It is predicted that the global surfactant market will grow by 4.5% from 2020 to 2025, in which cationic surfactants will encompass 6% of the global surfactant production [2]. Surfactants are used in industry and everyday life for cleaning, preservation, and as biocides. Cationic surfactants consist of a “fatty” tail and a positively charged head; the head can stick to oppositely charged surfaces and enhance the biocide effects [3]. Even though they have many positive effects in preventing the spread of viruses, overuse of cationic surfactant-based disinfectants can result in skin irritation and reproductive and respiratory problems. In addition to the negative effects on human health, cationic surfactants also represent a threat to the environment.

The classical approach for cationic surfactant quantification is two-phase titration based on color change [4]. This method is time-consuming, requires skilled personnel, lacks reproducibility, and uses toxic solvents. Methods such as fluorescence [5], luminescence [6], and solid phase extraction–ion chromatography with conductivity detection [7] have made progress in terms of reproducibility. On the other hand, these methods are time consuming, difficult to perform, and require expert personnel and organic solvents.

The employment of potentiometric chemical sensors [8] for the quantitative analysis of cationic surfactants represents a valuable substitution for existing methods. These devices can be divided into solid-state [9] and liquid membrane-type potentiometric sensors [10,11], which contain an ion-pair (ionophore) sensing element in the PVC-based liquid membranes. The ionophore is incorporated into the sensing membrane together with a plasticizer and a PVC [12,13,14]. Predicting the end-point break and modelling the titration system represents an additional challenge in the development of efficient surfactant sensors [14,15]. The ionophores usually consist of a surfactant-liked cation and a large negatively charged counter-ion. Ionophores are a crucial part of the sensing membrane since they affect sensor properties [16]. The ideal ionophore should have low solubility in water (inorganic solvent) and high solubility in the sensor membrane, which acts as an organic solvent (for the ionophore), as well as high stability. Suitable plasticizer selection has a crucial role in improving the sensor response and stability [10,11]. The addition of carbon-based nanomaterials to the sensing membrane can also improve the response properties [17,18]. Thus, to increase the sensitivity, lifetime, and signal stability, it is crucial to synthesize new ionophores and examine their properties.

This paper aims to describe the synthesis and characterization of a new 1,3-dioctadecyl-1*H*-imidazol-3-ium ion, which was employed as an ionophore for the construction of a potentiometric sensor for cationic surfactants, and to test the quantification ability in commercial products.

## 2. Results and Discussion

### 2.1. Synthesis and Characterization of the Ionophore

**Figure 1:**
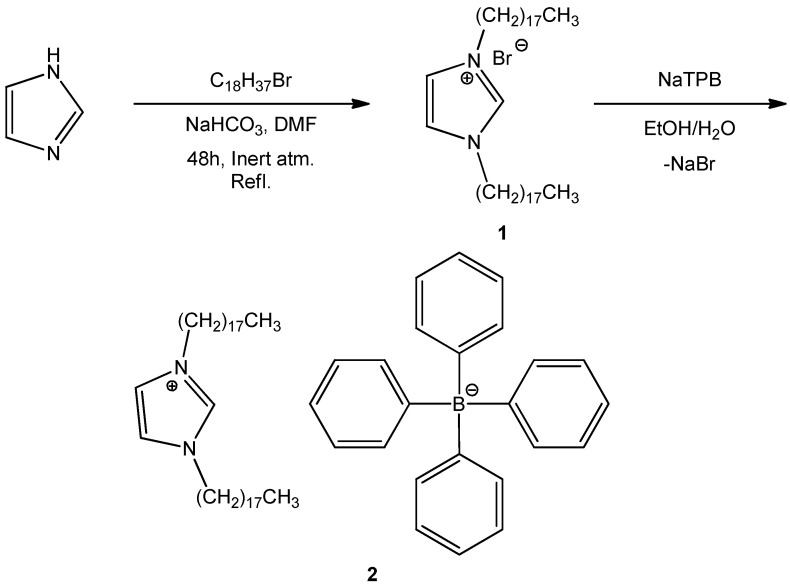
Synthesis of DODI–TPB ion-pair sensing complex (**2**) using quaternary alkyl ammonium salt 1,3-dioctadecyl-1*H*-imidazol-3-ium bromide (**1**).

Quaternary alkyl ammonium salt DODI-Br **1** was synthesized by reacting 1*H*-imidazole and 1-bromooctadecane (Figure 1). The reaction yield was established as 86.5%. The structure of synthesized compound **1** was confirmed by ^1^H- and ^13^C-NMR spectroscopy, mass spectrometry, and elemental analysis (Supplementary Materials Figures S1–S3).

The counter ion exchange of DODI-Br salt **1** was performed by a controlled reaction (NaTPB) to produce ionophore **2**, with a yield of 86.46% (Figure 1). DODI-TPB ionophore **2** was purified and incorporated into the PVC-based liquid ion sensor membrane. Insertion of the fabricated membrane in the Phillips ISE body electrode filled with electrolytes was the final step in surfactant sensor fabrication.

### 2.2. Sensor Characteristics

The characteristics of the DODI-TPB surfactant sensor were probed by a series of experimental tests followed by studies of the constructed sensor with real samples.

#### 2.2.1. Response Characteristics

The first step in characterization of the DODI-TPB surfactant sensor was measuring the potentiometric response towards cationic surfactants used in commercial product formulations, such as cetylpyridinium chloride (CPC), cetrimonium bromide, Hyamine 1622, and 1,3-didecyl-2-methylimidazolium chloride (DMIC). The latter is considerably more expensive and is used mostly in ionophore productions [19,20]. The response mechanism of the DODI-TPB surfactant sensor towards the cationic surfactants can be described by the modified Nernst equation:

(1) $$E=E_{0}+S\;log\;a_{CSurf^{+}}.$$

In Equation (1), E is the electromotive force, $E_{0}$ the constant potential term, S the slope of the sensor, and $a_{CSurf^{+}}\;$ the activity of the selected cationic surfactant.

Each surfactant was measured in a series of five independent tests and the obtained data are presented in Table 1. To fully evaluate the response information of the DODI-TPB surfactant sensor, four parameters for each surfactant were acquired: a slope of the surfactant sensor, correlation coefficient (R^2^), detection limit, and a useful linear concentration range. The DODI-TPB showed excellent response characteristics for all selected cationic surfactants. The slope was near-Nernstian, ranging from 57.2 mv/decade of activity (Hyamine 1622) up to 58.1 mv/decade of activity (CTAB) and 58.7 mv/decade of activity (CPC and DMIC). The correlation coefficient (R^2^) was very high, from 0.9961 (Hyamine 1622) up to 0.9997 (DMIC). The lowest detection limit value was achieved for DMIC at 0.9 × 10^−6^ M. The most useful linear concentration range was exhibited by DMIC, from 1.8 × 10^−6^ to 1.0 × 10^−4^ M; a similar range was observed for CPC, while CTAB and Hyamine 1622 had a useful linear concentration range from 4.8 × 10^−6^ and 5.1 × 10^−6^, respectively, to 1.0 × 10^−4^ M. The DODI-TPB surfactant sensor showed the best response characteristics towards DMIC and CPC cationic surfactants, although the sensor also exhibited good response characteristics for the other two studied cations (CTAB and Hyamine).

#### 2.2.2. Interference Study and pH Influence

Using interference studies, the influence of various inorganic and organic cations used in commercial product formulations on the response characteristics of the DODI-TPB surfactant sensor were evaluated. Although the sensor showed the best response characteristics with the DMIC and CPC cationic surfactants, the latter was used for further studies as it is considerably more economical.

The experiment was conducted by adding the CPC cationic surfactant to 10 selected interfering cationic ion solutions. The data were elaborated and selectivity coefficients were calculated using the Nikolskii–Eisenman equation and a fixed interference method (FIN) proposed by the IUPAC [21]. The chosen interfering ions for the study were as follows: NH_4_^+^, Na^+^, K^+^, Mg^2+^, Ca^2+^, 2-hydroxyethanaminium, tris(2-hydroxyethyl)ammonium, tetraethylammonium, benzyltrimethylammonium, and benzyltriethylammonium ions. The selectivity coefficients are presented in Table 2. The DODI-TPB surfactant sensor showed a good stability response towards CPC. For all ions, the calculated selectivity coefficients ranged from 1.7 × 10^−5^ to 9.1 × 10^−5^. For example, the highest discrimination of CPC was obtained towards ammonium and benzyltriethylammonium ions, with selectivity coefficients of 9.1 × 10^−5^ and 5.3 × 10^−5^, respectively (Entries 1 and 10), whereas the smallest selectivity coefficients were calculated for 2-hydroxyethanaminium, tris(2-hydroxyethyl)ammonium, and tetraethylammonium ions, with values of 1.7 × 10^−5^, 2.4 × 10^−5^, and 2.5 × 10^−5^, respectively (Entries 6, 7, and 8).

#### 2.2.3. Influence of pH

**Figure 2:**
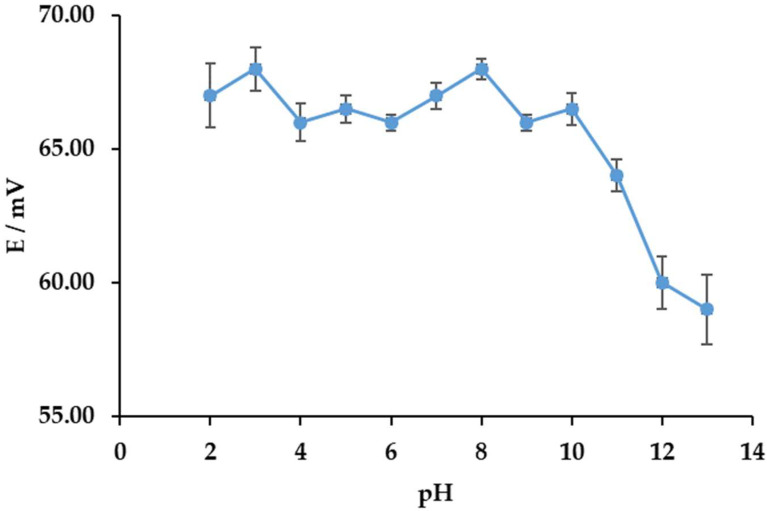
Investigation of the pH influence on the response characteristics of the DODI-TPB surfactant sensor to CPC (0.5 mM) (standard deviation errors bars included).

A pH range of 2 to 12 was used to observe the interfering effect on the DODI-TPB surfactant sensor towards CPC. No significant fluctuations of the signal were observed in the pH range from 2 to 10 (Figure 2). Thus, the proposed sensor could be used in a broad scale of different pHs, varying from acidic to basic.

### 2.3. Potentiometric Titration

The DODI-TPB surfactant sensor was used as an end-point indicator of the selected model sample solutions during potentiometric titrations.

#### 2.3.1. Titration of Model Samples

Direct potentiometric titrations of the cationic surfactant ($CSurf^{+}$), with anionic surfactant titrant ($ASurf^{-}$), results in the formation of a low-solubility ion-pair ($CSurf^{+}ASurf^{-}$).

This can be described by the following equation:

(2) $$CSurf^{+}+ASurf^{-}\Leftrightarrow CSurf^{+}ASurf^{-}$$

whereas dissociation of the formed ion-pair can be described by as the following equation:

(3) $$CSurf^{+}ASurf^{-}\Leftrightarrow CSurf^{+}+ASurf^{-}$$

The corresponding constant of the solubility product is:

(4) $$K_{sp}=a_{CSurf^{+}}a_{ASurf^{-}}$$

in which $a_{CSurf^{+}}$ and $a_{ASurf^{-}}$ are the activities of the selected surfactant cation and anion, respectively.

Prior to where the inflexion point (equivalence point) is reached, a decrease in the concentration of the cationic surfactant corresponds to a signal change according to Equation (1). Once the inflexion point is reached, the cationic surfactant is precipitated with anionic surfactant as a titrant. Its excess then dictates the electromotive force of the system.

The behavior of the sensor was tested by potentiometric titrations of model samples of cationic surfactants DMIC, CPC, CTAB, and Hyamine 1622. For titrations of the aforementioned cationic surfactants, two negatively charged ions were employed as titrants: an anionic surfactant dodecyl sulfate (DDS) and an organoboron anion tetraphenylborate (TPB).

**Figure 3:**
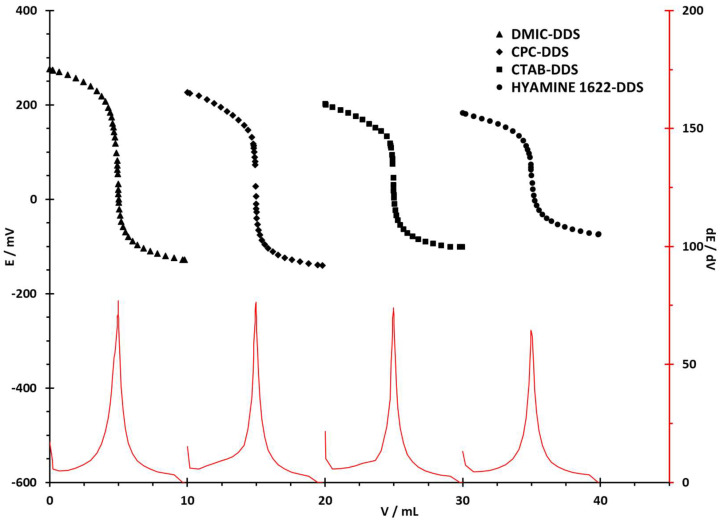
Potentiometric titration curves for 20 µmol of cationic surfactant with DDS (4 × 10^−3^ M). First derivatives are presented in full as the red lines below the titration curves, with corresponding values on the secondary y-axis.

**Figure 4:**
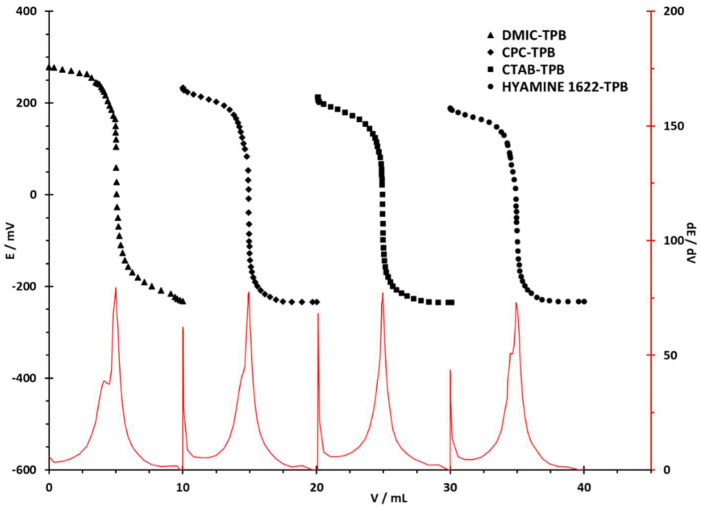
Potentiometric titration curves for 20 µmol of cationic surfactant with TPB (4 × 10^−3^ M). First derivatives are presented in full as the red lines below the titration curves, with corresponding values on the secondary y axis.

In conducted titrations, solutions containing 20 µmol of cationic surfactants were titrated. The titration curves with DDS and TPB as titrants and the DODI-TPB surfactant sensor as an end-point indicator are given in Figure 3 and Figure 4, respectively. The changes in potential for all four selected cationic surfactants were higher when the TPB solution was used as the titrant. For both titrants, the highest ΔE/mV was measured for DMIC: −510.0 mV for TPB and −406.1 mV for DDS; whereas the lowest was for Hyamine 1622: −422.1 mV for TPB and −257.4 mV for DDS, as presented in Table 3. The latter is due to the large differences in the ionic strengths of Hyamine 1622 and DMIC solutions, as well as to the affinities of DDS and TPB anions for the analyte. Thus, the strongest ion-pair was formed in the titration of DMIC with TPB, and the weakest in the Hyamine 1622 titration with DDS.

It is evident from the derivation curves (dE/dV) that the largest potential jump was recorded in the titration of DMIC with TPB (79.4) and the lowest in the titration of Hyamine 1622 with NaDDS (64.4). All titrations presented results with high accuracy and precision (RSD < 0.5%).

The accuracy of the DODI-TPB sensor was tested by the standard addition method, where a known amount of cationic surfactant (at two concentrations, 10 and 30 µmol) was added to the sample titrated with the DDS (4 × 10^−3^ M). Each measurement was independently repeated five times. The results of the potentiometric titration of CPC, CTAB, and Hyamine 1622 cationic surfactant solutions using the standard addition method are presented in Table 4. The added and determined values for each cationic surfactant were in good agreement with recoveries from 98.9 to 101.2 and an RSD below 0.5 %.

#### 2.3.2. Titration of Commercial Samples

After the DODI-TPB surfactant sensor was fully characterized and successfully applied as an end-point detector in the titration of model samples of cationic surfactants, it was used to quantify cationic surfactants in commercial samples. Since the COVID-19 pandemic began, the number of commercial products used as antiseptics and disinfectants has grown considerably. Several types of commercial products with declared cationic surfactant content were purchased from the local drugstore, including mouthwash, disinfectants for food industry applications, hand disinfectants, and disinfectants for hospital use. Typically declared cationic surfactants in these products were n-octyl-dimethyl-benzylammonium chloride, Hyamine 1622, CPC, CTAB, and methylbenzethonium chloride, with measured pH values between 5.5 and 9. The samples were titrated with corresponding amounts of DDS titrant and the DODI-TPB sensor was used as an end-point indicator.

A series of five tests were performed for each sample. Average values were compared with surfactant sensor data available in the quality control laboratory and the referent two-phase titration method (Table 5). RSD values for all measurements were below 0.5%. Data for all three methods showed good agreement. Even though further measurements should be performed, the DODI-TPB sensor has potential for use as a quality control tool for cationic surfactant quantification.

## 3. Materials and Methods

### 3.1. Reagents and Materials

Materials and reagents used for organic synthesis were all analytical grade chemicals, including 1-bromooctadecane, 1*H*-imidazole, and NaHCO_3_ (Sigma Aldrich, Darmstadt, Germany), with no additional purification during experiments.

Analytical grade cationic surfactants used for direct potentiometric response measurements and potentiometric titrations were as follows: CPC (Merck, Germany), Hyamine 1622 (Fluka, Buchs, Switzerland), CTAB (Fluka, Buchs, Switzerland), and DMIC (Merck, Munich, Germany).

Analytical grade TPB (Fluka, Buchs, Switzerland) was used for synthesis and for potentiometric titrations of selected cationic surfactants.

Analytical grade anionic surfactant DDS (Merck, Munich, Germany) was used for potentiometric titrations of selected cationic surfactants.

Membrane preparation analytical grade chemicals were a high molecular weight PVC (Sigma Aldrich), *o*-nitrophenyloctylether (*o*-NPOE) (Sigma Aldrich) plasticizer, and tetrahydrofuran (THF) (Merck, Munich, Germany).

Corresponding amounts of analytical grade NaOH and HCl (all from Kemika, Zagreb, Croatia) were used for pH adjustment.

For the preparation of all solutions, ultra-pure water was used.

A total of 12 commercial products, including disinfectants and antiseptics with declared cationic surfactant content, were purchased from the local drugstore as follows: mouthwash, disinfectants for food industry applications, hand disinfectant, and disinfectants for hospital use.

### 3.2. Synthesis of 1,3-Dioctadecyl-1H-imidazol-3-ium bromide (***1***)

The alkylation reaction of 1H-imidazole (0.24 g, 3.50 mmol) was performed under basic conditions by the addition of NaHCO_3_ (303.5 mg, 3.61 mmol) to anhydrous dimethylformamide (10 mL) and an excess of 1-bromooctadecane (4.22 g, 12.65 mmol). The solution was stirred at 90 °C under an inert nitrogen atmosphere for 48 h. The progress of the reaction was followed by TLC (DCM:methanol = 10:0.25). The product was washed with methanol and hexane three times, followed by flash column purification (DCM:methanol = 10:0.25) and drying under a dynamic vacuum at room temperature for 4 h. The desired bisalkylated product **1** (1.98 g, 3.03 mmol) was obtained at a yield of 86.5%. A detailed characterization of DODI-Br by MS, ^1^H NMR, ^13^C NMR spectroscopy, and elementary analysis is provided in the Supplementary Materials (Figures S1–S3).

### 3.3. Characterization of 1,3-Dioctadecyl-1H-imidazol-3-ium bromide

^1^H NMR spectra were recorded at 600 MHz and ^13^C NMR spectra at 150.9 MHz using a Bruker AV600 spectrometer (Bruker BioSpin GmbH, Rheinstetten, Germany) at Ruđer Bošković Institute. Chemical shifts were referenced to the residual solvent peak (DMSO-d6) with SiMe_4_ as the internal standard.

Spectroscopic information on the molecular ions was obtained through the API 2000 LC-ESI-MS/MS (Applied Biosystems, Foster City, CA, USA) in q1 ms scan mode.

For the elemental analysis, a PerkinElmer 2400 CHNS/O Series II System was used (PerkinElmer Inc., Waltham, MA, USA).

### 3.4. Preparation of DODI-TPB Surfactant Sensor

To prepare the sensing ion-pair DODI-TPB, an ethanol/water solution (volume ratio 2:1) of 1,3-dioctadecyl-1*H*-imidazol-3-ium bromide (**1**) and an aqueous solution of NaTPB (0.05 M) were separately heated to 50 °C. Then, 10 mL of hot Na-TPB solution was slowly added to 30 mL of hot DHBI-Br solution in ethanol/water containing an equimolar quantity of 1,3-dioctadecyl-1*H*-imidazol-3-ium bromide. The solution became opaque and the reaction mixture was further stirred and slowly heated. At 70 °C, the mixture became transparent, and the white precipitate appeared at 80 °C. The white precipitate was removed. After evaporation of the ethanol, the crude DODI-TPB complex was washed with deionized water, filtrated, and dried at 80 °C to a constant mass. This preparation of the ion-pair was used for sensor membrane fabrication.

One percent of the prepared DODI-TPB ion-pair was added to a mixture of high molecular weight PVC and a plasticizer o-NPOE (1:2). After sonication, 0.1 g of the obtained cocktail was added to 2 mL of THF and sonicated for a further 10 to 15 min. The cocktail was poured into a glass mold, dried, and then cut into smaller sensor membranes. The sensor membrane was installed in the Philips electrode body IS-561 (Supelco, Bellefonte, PA, USA) filled with NaCl (3 M).

### 3.5. Measuring Setup

The Metrohm 794 Basic Titrino paired with the Metrohm 781 pH meter, and a corresponding stirrer (Metrohm, Herisau, Switzerland), were used for response measurements, interference measurements, and pH studies.

The Metrohm 808 Titrando with a stirrer and the Metrohm Tiamo software 2.1 were used for potentiometric titrations.

The Metrohm silver/silver (I) chloride electrode with potassium chloride (3 M) electrolyte solution was used as a reference electrode for all measurements.

### 3.6. Procedure

All measurements were carried out with a two-electrode system, a DODI-TPB surfactant sensor, and a referent Ag/AgCl electrode. Deionized water was used in all experiments.

#### 3.6.1. Sensor Characterization

Direct potentiometry was used for the characterization of the DODI-TPB surfactant sensor. The response characteristics were measured by the incremental addition of selected cationic surfactants (DMIC, CPC, CTAB, and Hyamine 1622) in deionized water. The final concertation range of selected cations in the logarithmic scale ranged from −2 to −8. The potential of the prepared sensor was tested in the usual concentration ranges found in commercial products and in environmental water samples.

To observe the response of the surfactant sensor in the presence of various cations used in commercial product formulations, interference studies were conducted. The concentrations of solutions of interfering cations were adjusted to 0.01 M and CPC was incrementally added to the interfering ion solutions. The CPC concentrations on the logarithmic scale ranged from −2 to −8. Potentiometric selectivity coefficients were calculated according to the IUPAC fixed interference method [21].

The impact of pH on the response of the DODI-TPB surfactant sensor when titrating the CPC solution (0.5 mM) ranged from pH 2 to 12. The pH values were adjusted with NaOH (0.5 M) and HCl (0.5 M) solutions.

All response measurements were carried out at room temperature. The surfactant sensor was stored in deionized water.

#### 3.6.2. Potentiometric Titrations

For potentiometric titrations with the DODI-TPB surfactant sensor, the Metrohm measuring system was used. The dynamic equivalence point titration (DET) mode was used for all conducted titrations, with a 5 mV/min signal drift. The titrant was added in steps at variable volumes. The slope of the titration curve was dependent on the increments of added titrant. After each measurement, the electrode system was washed with deionized water. All titration measurements were carried out at room temperature.

The concentration of cationic surfactants in commercial samples of disinfectants and antiseptics was measured by potentiometric titrations using DDS (4 × 10^−3^ M) as a titrant and the DODI-TPB surfactant sensor as an end-point indicator.

## 4. Conclusions

The new cationic surfactant 1,3-dioctadecyl-1*H*-imidazol-3-ium bromide (DODI-Br) was successfully characterized by NMR, LC-MS, and elemental analysis. After DODI-Br was converted to the DODI-TPB ion-pair via the counter-ion exchange reaction, it was used to fabricate the DODI-TPB surfactant sensor. The DODI-TPB surfactant sensor was successfully characterized and showed excellent response characteristics to cationic surfactants DMIC, CPC, CTAB, and Hyamine 1622, with near-Nernstian slopes. The best slope values were obtained for DMIC and CPC (58.7 mV/decade of activity), whereas the lowest detection limits were observed for DMIC (0.9 × 10^−6^ M) and CPC (2.1 × 10^−6^ M). The broadest useful linear concentration range was shown by DMIC (1.80 × 10^−6^ to 1.0 × 10^−4^ M).

The DODI-TPB surfactant sensor did not show deviations in the response signal when exposed to interfering cations usually present in commercial samples. Furthermore, pH had no influence on the response signal in the pH range 2 to 10. Excellent sigmoidal curves with well-defined inflexion points and broad signal change were obtained for the potentiometric titration of cationic surfactants (DMIC, CPC, CTAB, and Hyamine 1622) with DDS and TPB. Titration curves measured with TPB as a titrant had a higher signal change; however, first derivative values for both titrants were similar. The standard addition method was successfully applied with excellent recovery rates ranging from 98.9 to 101.2, at two concentrations. The results of the potentiometric titrations of 12 disinfectants and antiseptic products were in good agreement with the results obtained by the two-phase titration standard method and commercial surfactant sensor. This new surfactant sensor represents a low-cost and simple alternative to existing methods for cationic surfactant analysis and could be used as a quality control tool in product formulations. Additionally, a low LOD could enable the use of proposed sensor for environmental applications, such as the detection of low cationic surfactant concentrations in waters and wastewaters.

## Figures and Tables

**Table 1:** Response characteristics of the DODI-TPB surfactant sensor to DMIC, CPC, CTAB, and Hyamine 1622 (mean values ± 95% confidence limits).

Parameters	DMIC	CPC	CTAB	Hyamine 1622
Slope (mV/decade)	58.7 ± 0.5	58.7 ± 0.9	58.1 ± 1.2	57.2 ± 1.4
Correlation coefficient (R^2^)	0.9997	0.9995	0.9993	0.9961
Detection limit (M)	0.9 × 10^−6^	1.2 × 10^−6^	2.3 × 10^−6^	3.5 × 10^−6^
Useful linear conc. range (M)	1.8 × 10^−6^ to 1.0 × 10^−4^	2.1 × 10^−6^ to 1.0 × 10^−4^	4.8 × 10^−6^ to 1.0 × 10^−4^	5.1 × 10^−6^ to 1.0 × 10^−4^

**Table 2:** Interfering influence of the selected inorganic and organic cations towards CPC.

Entry	Interfering Cations	$K_{Cat_{i.}^{+}}^{pot}$
1	ammonium	9.1 × 10^−5^
2	sodium	3.6 × 10^−5^
3	potassium	5.1 × 10^−5^
4	magnesium	3.7 × 10^−5^
5	calcium	3.9 × 10^−5^
6	2-hydroxyethanaminium	1.7 × 10^−5^
7	tris(2-hydroxyethyl)ammonium	2.4 × 10^−5^
8	tetraethylammonium	2.5 × 10^−5^
9	benzyltrimethylammonium	4.8 × 10^−5^
10	benzyltriethylammonium	5.3 × 10^−5^

**Table 3:** Response characteristics for the DODI-TPB surfactant sensor to DMIC, CPC, CTAB, and Hyamine 1622 (mean values ± 95% confidence limits).

Cationic Surfactant	Titrant			
	DDS	TPB	DDS	TPB
	Δ*E*/mV		d*E*/d*V*	
DMIC	−406.1	−510.0	77.0	79.4
CPC	−366.6	−466.7	76.4	77.5
CETAB	−302.9	−447.4	74.0	77.3
Hyamine 1622	−257.4	−422.1	64.4	73.0

**Table 4:** Potentiometric titration of CPC, CTAB, and Hyamine 1622 cationic surfactant solutions using the standard addition method. DDS was used as a titrant (4 × 10^−3^ M) (mean values ± 95% confidence limits).

Cationic Surfactant	*n* (Added)/µmol	*n* (Found) */µmol	Recovery/%	RSD/%
CPC	30	30.04 ± 0.04	100.1	0.34
	10	10.09 ± 0.05	100.9	0.29
CTAB	30	30.12 ± 0.05	100.4	0.21
	10	9.89 ± 0.02	98.9	0.36
Hyamine 1622	30	29.92 ± 0.06	99.7	0.32
	10	10.12 ± 0.04	101.2	0.41

* average on 5 determinations.

**Table 5:** Cationic surfactant content in commercial samples obtained by potentiometric titrations with DDS (4 × 10^−3^ M), with the DODI-TPB sensor as an end-point indicator, in addition to a comparison with existing referent methods.

Product	DODI-TPB Sensor		Surfactant Sensor [17]		Two-Phase Titration [4]
	%	RSD (%)	%	RSD (%)	%
1	4.621	0.32	4.637	0.37	4.594
2	4.342	0.29	4.363	0.39	4.393
3	4.854	0.42	4.865	0.41	4.882
4	4.255	0.38	4.338	0.41	4.315
5	0.112	0.38	0.114	0.41	0.116
6	0.004	0.49	0.004	0.42	0.005
7	0.242	0.32	0.237	0.44	0.249
8	0.086	0.41	0.081	0.35	0.087
9	0.094	0.36	0.089	0.39	0.087
10	0.054	0.32	0.056	0.34	0.053
11	0.046	0.34	0.046	0.39	0.041
12	0.116	0.34	0.112	0.39	0.114

Aaverage of 5 determinations.

## Data Availability

The data presented in this study are available in Supplementary Materials.

## Supplementary Materials

The following supporting information can be downloaded at: https://www.mdpi.com/article/10.3390/s22239141/s1, Figures S1 to S3: Figure S1. ^1^H NMR of 1,3-dioctadecyl-1*H*-imidazol-3-ium bromide (**1**). Figure S2. ^13^C APT NMR of 1,3-dioctadecyl-1*H*-imidazol-3-ium bromide (**1**). Figure S3. Positive ESI-MS/MS Q1 scan for 1,3-dioctadecyl-1*H*-imidazol-3-ium bromide (**1**); infusion 10 µL min^−1^ at concentration 2.5 ng µL^−1^.
