# Supplementary material for: The 1,3-Dioctadecyl-1H-imidazol-3-ium Based Potentiometric Surfactant Sensor for Detecting Cationic Surfactants in Commercial Products
Source: Sensors (Basel). 2022 Nov 25;22(23):9141. doi: 10.3390/s22239141 (PMC9739083; doi:10.3390/s22239141)
Supplement: Supplementary file 1 [file sensors-22-09141-s001.zip › sensors-1990192-supplementary.pdf]

Article

The 1,3-Dioctadecyl-1*H*-imidazol-3-ium Based Potentiometric Surfactant Sensor for Detecting Cationic Surfactants in Commercial Products

Nikola Sakač [1,*], Dubravka Madunić-Čačić [1,2], Dean Marković [3], Bartolomeo Della Ventura [4], Raffaele Velotta [4], Anita Ptiček Siročić [1], Brunislav Matasović [5], Nikolina Sermek [5], Bojan Đurin [6], Bojan Šarkanj [7] and Marija Jozanović [5,*]

[1] Faculty of Geotechnical Engineering, University of Zagreb, 42000 Varaždin, Croatia
[2] Saponia Chemical, Pharmaceutical and Foodstuff Industry, Inc., 31000 Osijek, Croatia
[3] Department of Biotechnology, University of Rijeka, 51000 Rijeka, Croatia
[4] Department of Physics "E. Pancini", Università Di Napoli Federico II, 80126 Napoli, Italy
[5] Department of Chemistry, University of Osijek, 31000 Osijek, Croatia
[6] Department of Civil Engineering, University North, 42000 Varaždin, Croatia
[7] Department of Food Technology, University North, 48000 Koprivnica, Croatia
* Correspondence: nikola.sakac@gfv.unizg.hr (N.S.); mjozanovic@kemija.unios.hr (M.J.); Tel.: +385-915830336 (N.S.); +385-996865716 (M.J.)

Table of contents

S1. ^{1}H NMR of 1,3-dioctadecyl-1*H*-imidazol-3-ium bromide (**1**)

S2. ^{13}C APT NMR of 1,3-dioctadecyl-1*H*-imidazol-3-ium bromide (**1**).

S3. Positive ESI-MS/MS Q1 scan for 1,3-dioctadecyl-1*H*-imidazol-3-ium bromide (**1**); infusion 10 μL min^{-1} at a concentration of 2.5 ng μL^{-1}.

1,3-dioctadecyl-1*H*-imidazol-3-ium bromide (1)

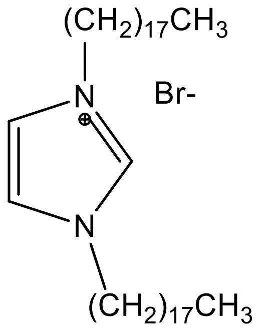

The alkylation reaction of 1*H*-imidazole (0.24 g, 3.50 mmol) was performed under basic conditions by the addition of $NaHCO_3$ (303.5 mg, 3.61 mmol) in anhydrous dimethylformamide (10 mL) and an excess of 1-bromooctadecane (4.22 g, 12.65 mmol). The reaction progress was followed by TLC (DCM:methanol = 10:0.25), and the reaction was carried out under reflux for 48 h in an inert nitrogen atmosphere. NaBr salt was precipitated with hexane and the crude product **1** was obtained by filtration. Further purification by flash chromatography (DCM:methanol = 10:0.25) gave the desired bisalkylated ionic liquid **1** (0.941 g, 1.66 mmol) at a yield of 86.46%.

White powder, 86.46% yield (1.98 g, 3.03 mmol); m.p.= 81.4-83.9°C; R_f = 0.52 (DCM:methanole = 10:0.25); **^{1}H NMR** (300 MHz; $CDCl_3$): δ 10.57 (1H, s, H-C(2)), 7.41 (2H, d, J=1.5 Hz, H-C(4), H-C(5)), 4.37 (4H, t, J=7.5 Hz, H2-C(1')), 1.95-1.90 (4H, m, H2-C(2')), 1.33-1.25 (60H, m, H2-C(3'-17')), 0.88 (6H, t, J=6.5 Hz, H3-C(18')); **^{13}C NMR** (75,48 MHz; $CDCl_3$): δ 137.3, 121.8, 50.1, 31.9, 30.3, 29.7 (5C atoms), 29.65 (2C atoms), 29.6, 29.5, 29.4, 29.3, 29.0, 26.2, 22.7, 14.1 **IR** (KBr, cm^{-1}): 3450, 3100, 2900, 2850, 2050, 100, 1550, 1450, 1150. **MS-CI** (NH_3): 574, 485, 417, 381, 321, 283, 255, 183, 121, 69. **Elem. anal.** (calc. for $C_{39}H_{71}BrN_2$): C 71.63; H 11.87; Br, 12.22; N 4.28; found: C 71.84; H 12.01; Br, N/A; N 4.30.

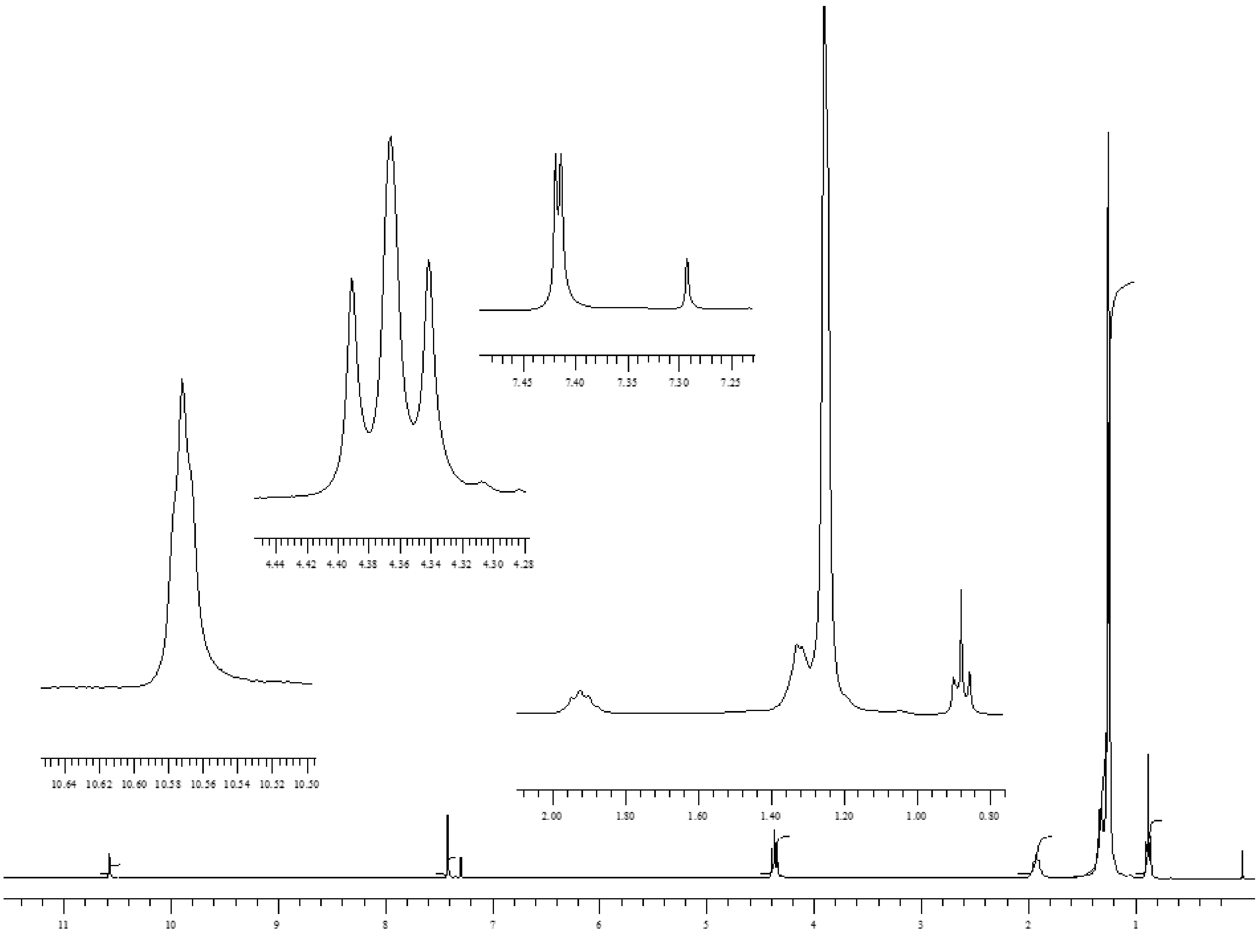

10.64 10.62 10.60 10.58 10.56 10.54 10.52 10.50
4.44 4.42 4.40 4.38 4.36 4.34 4.32 4.30 4.28
7.45 7.40 7.35 7.30 7.25
2.00 1.80 1.60 1.40 1.20 1.00 0.80
11 10 9 8 7 6 5 4 3 2 1

Figure S1. ¹H NMR of 1,3-dioctadecyl-1*H*-imidazol-3-ium bromide (**1**).

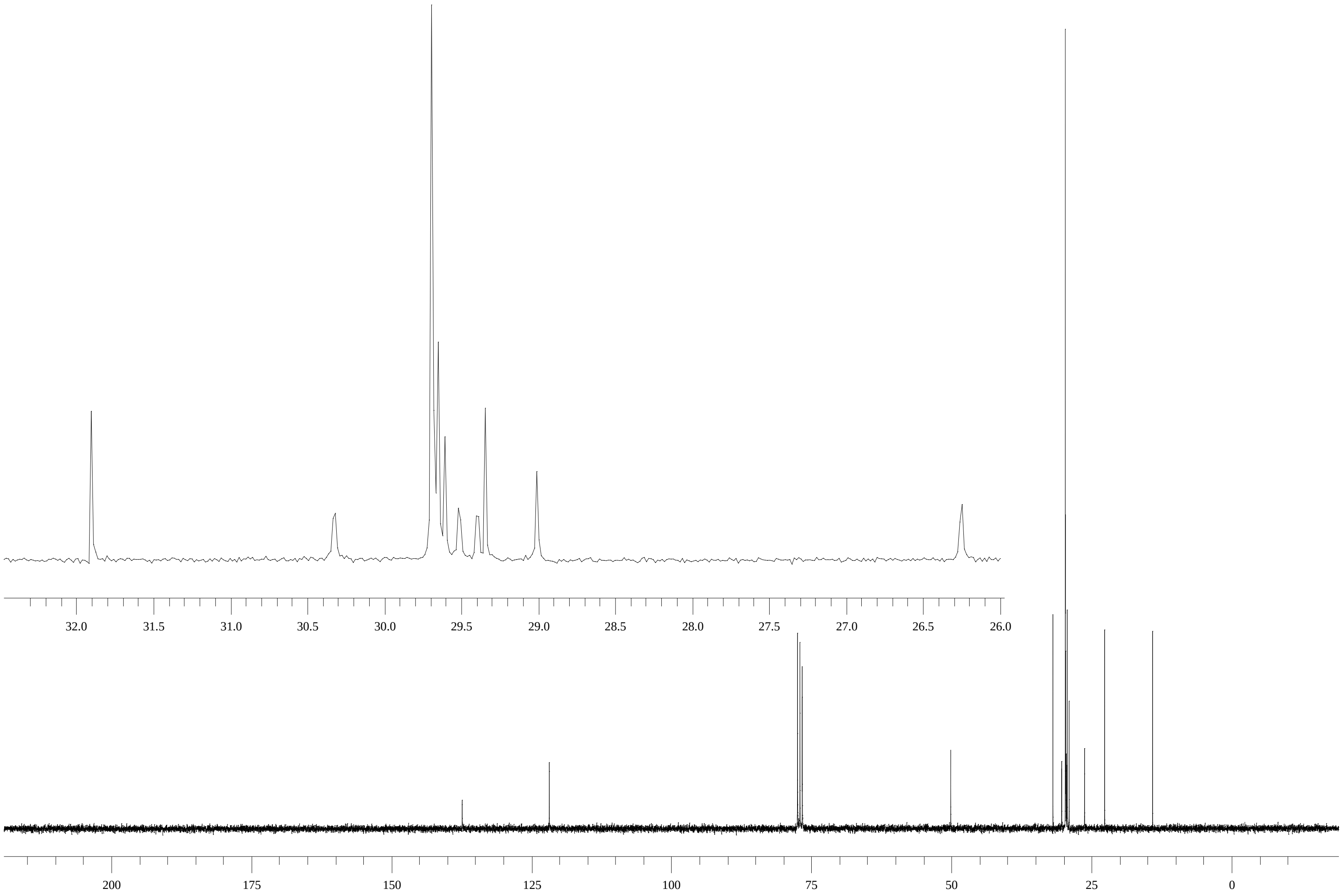

Figure S2. ^{13}C NMR of 1,3-dioctadecyl-1*H*-imidazol-3-ium bromide (**1**).

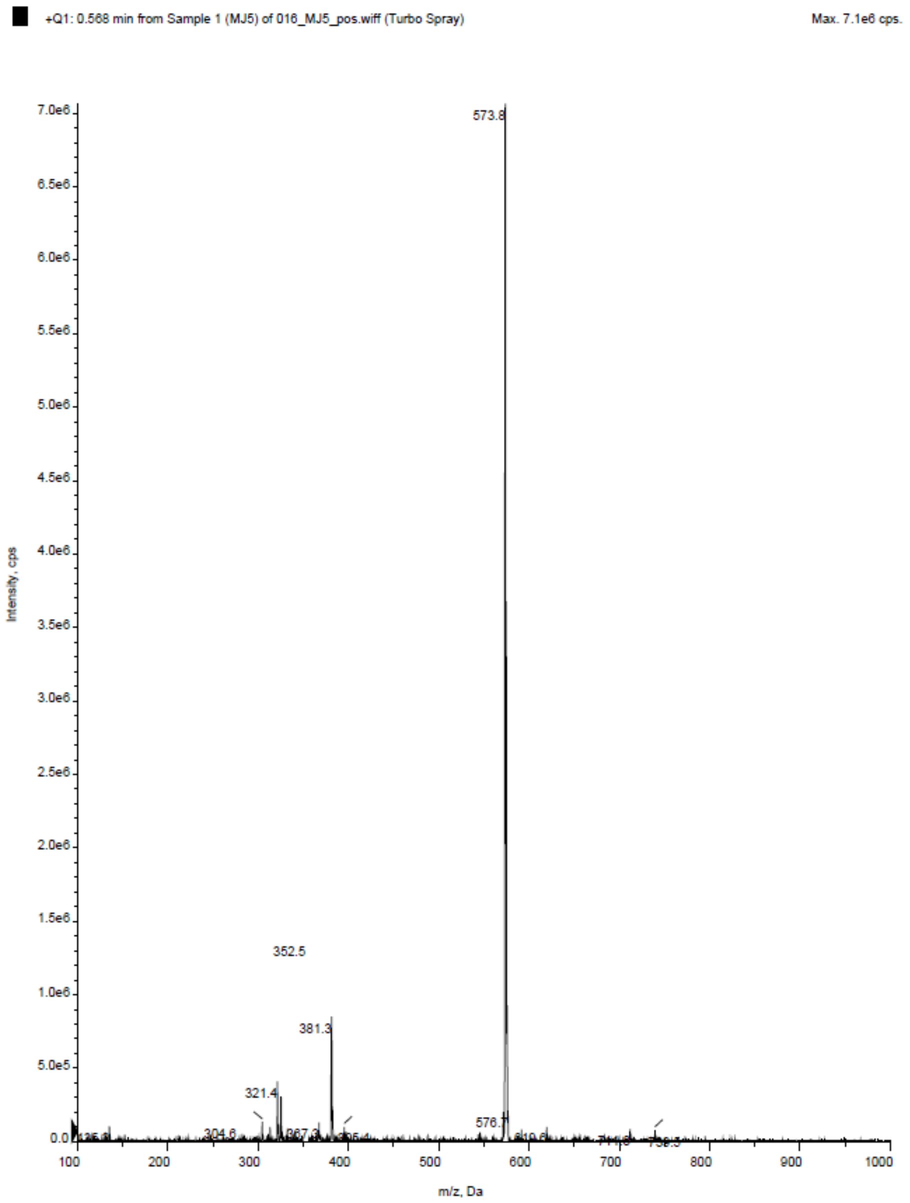
+Q1: 0.568 min from Sample 1 (MJ5) of 016_MJ5_pos.wiff (Turbo Spray)
Max. 7.1e6 cps.
Intensity, cps
m/z, Da
573.8
352.5
381.3
321.4
576.1
304.6

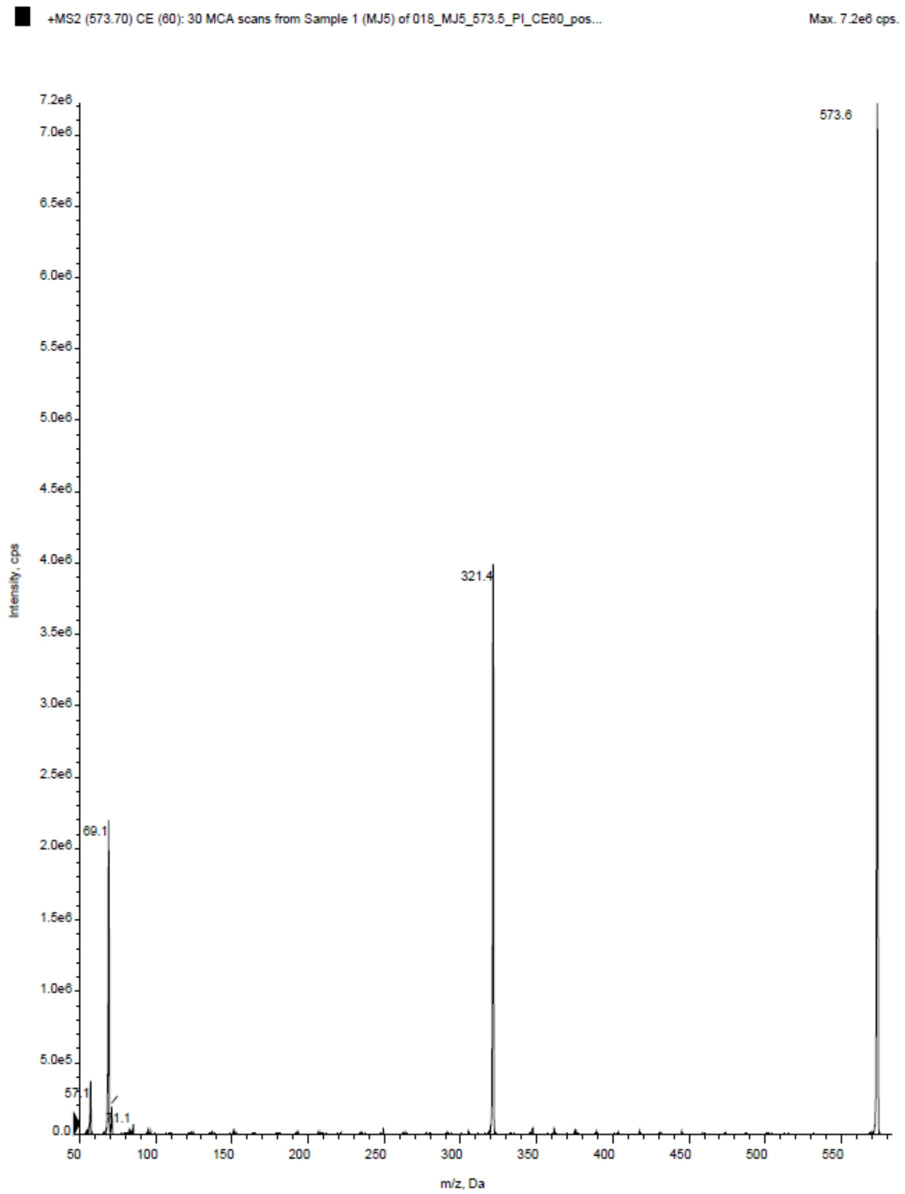
+MS2 (573.70) CE (60): 30 MCA scans from Sample 1 (MJ5) of 018_MJ5_573.5_PI_CE60_pos...
Max. 7.2e6 cps.
573.6
321.4
69.1
57.1
1.1
Intensity, cps
m/z, Da

Figure S3. Positive ESI-MS/MS Q1 scan for 1,3-dioctadecyl-1*H*-imidazol-3-ium bromide; infusion 10 μL min^{-1} at concentration 2.5 ng $μL^{-1}$.
